# Staged conversion after circular ring external fixation in adult open long-bone fractures: A systematic review^☆^

**DOI:** 10.1016/j.jcot.2026.103398

**Published:** 2026-03-06

**Authors:** Domenico Fenga, Daniel Pérez-Prieto, Gabriele Giuca, Paolo Rizzo, Biagio Zampogna, Danilo Leonetti

**Affiliations:** a Section of Orthopaedics and Traumatology, Department of Biomedical Sciences and Morphological and Functional Images, University of Messina, 98122, Messina, ME, Italy; b Hospital del Mar, Barcelona, Spain, Autonomous University of Barcelona, Barcelona, Spain; c Department of Orthopaedic and Trauma Surgery, Università Campus Bio-Medico Di Roma, Rome, Italy; d Research Unit of Orthopaedic and Trauma Surgery, Fondazione Policlinico Universitario Campus Bio-Medico, Rome, Italy

**Keywords:** Open fractures, External ring fixator, Internal fixation, Ilizarov, Pin tract infection, Fracture healing, Gustilo-anderson classification

## Abstract

**Background:**

Circular ring external fixation is frequently used in high-energy open long-bone fractures, yet prolonged frame retention is associated with pin-tract morbidity and patient burden. Staged conversion to internal fixation is therefore attractive, but concerns persist regarding deep infection and nonunion. We systematically reviewed the evidence on outcomes and timing of staged conversion following circular ring fixation in adult open long-bone fractures.

**Methods:**

This review followed PRISMA 2020 guidelines and was registered in PROSPERO (CRD42021023282). PubMed, Embase, Cochrane Library, and Scopus were searched from inception to December 2025. We included studies of adult open long-bone fractures managed with circular ring external fixation, comparing definitive ring fixation versus staged conversion to internal fixation or reporting outcomes after conversion. Owing to clinical and methodological heterogeneity, a narrative synthesis was performed. Primary outcomes were reported deep infectious complications (including fracture-related infection when explicitly defined) and union.

**Results:**

Eight studies (303 patients) were included; however, only six acute traumatic open-fracture cohorts contributed to the primary synthesis, while two reconstructive/infected nonunion series were considered contextual only. Across the limited acute-trauma data, staged conversion was associated with fewer frame-related complications in some cohorts, but any effect on deep infectious complications and union remained uncertain because of inconsistent definitions, small sample sizes, and confounding by indication.

**Conclusions:**

The current evidence base is dominated by small, retrospective and methodologically heterogeneous studies. In acute traumatic open long-bone fractures treated with circular ring fixation, staged conversion after soft-tissue stabilization may reduce frame-related morbidity but whether it influences deep infection or union remains uncertain. Present data should therefore be interpreted as low-certainty observations rather than reliable comparative evidence.

**Level of evidence:**

Level IV

## 1 Introduction

Open fractures, defined by a break in the bone that communicates with the external environment, are known for their propensity toward infection and prolonged healing.^1^ Fracture-related infection (FRI) remains one of the most serious complications after open fractures and may occur in up to one-quarter of such injuries, particularly in high-grade Gustilo–Anderson type III fractures. Standardized FRI definitions have only recently been established, which has historically made comparison between studies difficult.^2^^,^^3^ Their management typically demands prompt stabilization and cautious soft tissue handling to mitigate infection risk and prevent additional soft tissue compromise.^4^ External fixation with circular ring constructs is integral to damage-control management of severe open fractures, as it provides rapid stabilization, preserves the soft-tissue envelope and facilitates repeated debridement and staged reconstruction when needed. Nevertheless, prolonged frame use is limited by pin-site complications, patient discomfort, and constraints on joint motion, leading many surgeons to favor a staged protocol with later conversion to intramedullary nailing or internal plate fixation once the soft-tissue envelope has recovered.^5^ Despite the recognized benefits, prolonged use of external fixation carries notable drawbacks. Pin tract infections, discomfort, and limitations on patient mobilization are well-documented.^6^^,^^7^ Accordingly, some clinicians advocate for staged protocols that involve converting from external to internal fixation, such as intramedullary nailing or plating, after the acute risk of contamination has subsided.^8^ Theoretical advantages of this approach include enhanced mechanical stability, potentially lower infection rates once the soft tissues have recovered and improved functional outcomes due to earlier mobilization.^9^ Still, concerns linger about placing internal hardware into an area previously compromised by open injury or contaminated pin sites.^10^ The timing of such a conversion is pivotal; too early and the risk of deep infection may increase, yet waiting too long might permit complications such as persistent pin tract infections, delayed union, or limited joint motion.^11^^,^^12^ Prior studies underscore this tension, with some demonstrating substantial improvements in function and infection rates after conversion,^13^ whereas others highlight increased operative demands or uncertain risk profiles.^14^ Prior cohort studies suggest that both premature conversion before adequate debridement/coverage and very late conversion after prolonged frame retention may be associated with higher complication rates; however, reported thresholds vary widely and are strongly confounded by injury severity and soft-tissue status. These observations, however, derive from heterogeneous, mostly single-center series and remain validated in larger, prospective cohorts.^12^^,^^15^^,^^16^ The clinical implications are significant: if conversion to internal fixation is shown to reliably reduce complications and foster better functional recovery, guidelines may shift to encourage routine staged procedures for open fractures. On the other hand, if data remains inconsistent, surgeons might continue using external fixation in a more prolonged fashion, particularly for patients with poor soft tissue envelope conditions or comorbidities that raise infection risk. Against this background, we undertook a systematic review to synthesize the evidence on staged conversion from circular ring external fixation to internal fixation in adults with acute traumatic open long-bone fractures, while reporting reconstructive or infected nonunion series separately given their distinct biological and infectious profiles.

## 2 Methods

This systematic review followed the Preferred Reporting Items for Systematic Reviews and Meta-Analyses (PRISMA) guidelines. The review protocol was registered with PROSPERO (Registration Number: CRD42021023282).

### 2.1 Search strategy

A comprehensive search was carried out in four databases, PubMed/MEDLINE, EMBASE, Cochrane Library, and Scopus, to locate studies published from database inception until December 2025. The search combined Medical Subject Headings (MeSH) and keywords such as “open fractures,” “external ring fixator,” “Ilizarov,” “internal fixation,” “conversion,” and “complications.” Results were restricted to articles in English. Additional sources, including ClinicalTrials.gov and major orthopedic conference proceedings, were examined to identify grey literature and minimize publication bias. Reference lists from relevant articles were also reviewed.

### 2.2 Inclusion and exclusion criteria

We included comparative and observational studies enrolling adults (≥18 years) with acute traumatic open long-bone fractures in which the index external fixation construct was a circular ring system (Ilizarov-type frames, Taylor Spatial Frame and related ring-based constructs), and that reported at least one outcome after either continued ring fixation or staged conversion to internal fixation. Studies focused primarily on infected nonunion, failed prior fixation, distraction osteogenesis or other reconstructive indications were not included in the primary analytic set; when retained for contextual completeness, they were reported separately and were not used to support comparative inferences regarding acute trauma.

### 2.3 Study selection and data extraction

After removal of duplicates, two reviewers independently screened titles and abstracts to identify potentially eligible studies. Full texts were then retrieved and assessed in duplicate against predefined inclusion/exclusion criteria. Any disagreement at either stage was resolved by discussion; when consensus was not reached, a third reviewer adjudicated. Data extraction was performed independently by two reviewers using a standardized, piloted extraction form. Extracted variables included: study characteristics (authors, year, country, design, sample size, follow-up); patient and injury details (age, sex, limb, fracture location, Gustilo–Anderson grade and/or AO/OTA classification when reported); external fixation strategy with emphasis on circular/ring fixation (Ilizarov, Taylor Spatial Frame, and hybrid ring constructs), including indications and duration; conversion strategy (yes/no), internal fixation modality (e.g., IMN, plate), and timing of conversion (as reported by each study). Outcomes were extracted as defined in the primary studies, including fracture-related infection/deep infection, pin-tract infection, union/nonunion/delayed union, reoperation, and functional scores. When definitions varied across studies, we recorded the original definition verbatim and addressed the resulting heterogeneity in the synthesis.

### 2.4 Operational outcome definitions

Because outcome terminology varied substantially across studies, we extracted authors’ original definitions verbatim whenever reported. When studies did not provide explicit criteria, outcomes were categorized only at a broad descriptive level (e.g., “reported deep infectious complication,” “reported delayed healing,” or “reported nonunion”) without imposing strict diagnostic thresholds intended to replicate modern consensus criteria. Importantly, historical reports of deep infection were not assumed to be equivalent to contemporary fracture-related infection (FRI) unless the study explicitly used criteria consistent with modern FRI frameworks. This approach was chosen to minimize misclassification while acknowledging major cross-study heterogeneity.

### 2.5 Risk of bias and quality assessment

Two reviewers independently assessed methodological quality using the Newcastle–Ottawa Scale (NOS) after calibration. Domain-level scores for each study are reported in Supplementary Table S1; disagreements were resolved by consensus.

### 2.6 Data synthesis and statistical analysis

Given substantial clinical and methodological heterogeneity (fracture sites, Gustilo grades, fixator constructs, conversion criteria and outcome definitions), we did not perform a meta-analysis. Instead, we conducted a structured narrative synthesis, presenting study-level results as ranges and medians, and separating comparative data from single-arm cohorts. Where reported, conversion timing was extracted exactly as described by the original authors and summarized descriptively, without imposing uniform temporal cutoffs or deriving practice guidance from heterogeneous reports. We prioritized ring fixator studies and highlighted ring-specific considerations.

## 3 Results

### 3.1 Study selection

**Fig. 1:**
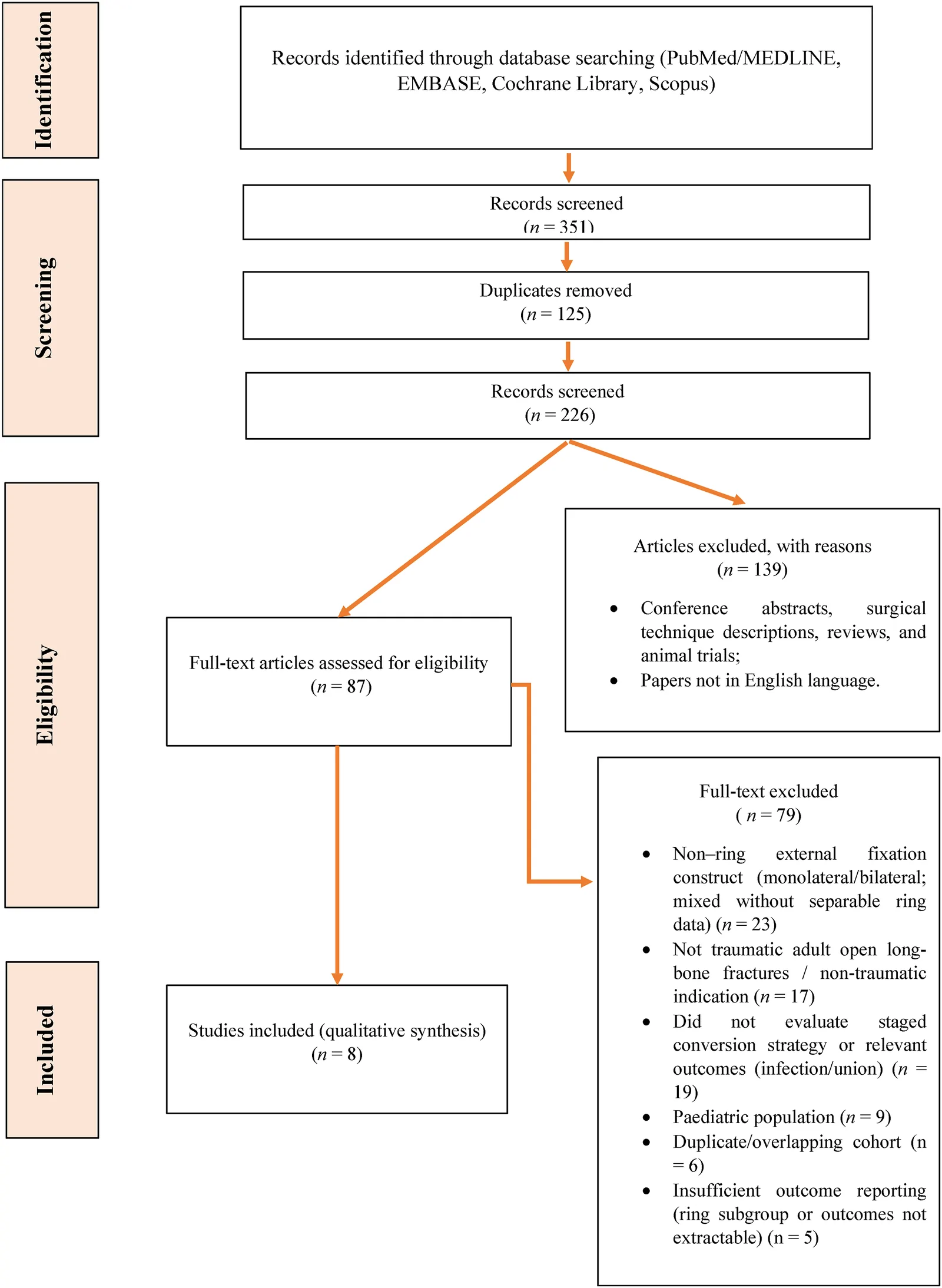
PRISMA flow-diagram.

Database searches yielded 351 records; 125 duplicates were removed, leaving 226 titles/abstracts screened. After excluding 139 records, 87 full texts were assessed; 79 were excluded with prespecified reasons (Fig. 1). The final review comprised 8 included studies (303 patients), of which 6 addressed acute traumatic open long-bone fractures and formed the primary analytic set, while 2 reconstructive or infected nonunion cohorts were retained only as contextual evidence and are presented separately, because their biology, indications and baseline infection risk differ substantially from acute trauma cohorts (Table 1 A-B).

**Table 1:** Included Studies. (A) Acute traumatic adult open long-bone fracture cohorts; (B) Reconstructive/infected nonunion cohorts.

(A)										
	First Author (Year)	Design	N	Gustilo/AO	External Fixator	Converted to IF?	IF Type	Union Rate	Infection Rate	Functional Outcome
1	Zhang (2020)^17^	Retrospective	8	IIIB/C	Ilizarov + Flap	X	–	100%	1 case minor	ASAMI: Excellent
2	Sala (2013)^18^	Retrospective	52	Open long bone	Taylor Spatial	X	–	91%	Low	ASAMI: Excellent
3	Dhar (2008)^10^	Retrospective	28	Polytrauma	Ilizarov	✓	–	100%	Controlled	ASAMI: Excellent
4	Bacon (2008)^19^	Retrospective	42	Type C pilon	Ilizarov vs ORIF	Parallel groups	Plates	NS diff	NS diff	No score used
5	Jin (2022)^20^	Retrospective	88	Arterial limb	Mixed EF (circular ring subgroup extracted)	X	–	91.8%	3 deep inf	LEFS improved
6	Lim (2020)^21^	Retrospective	45	Open long bone	Mono/Circular EF (circular ring subgroup extracted)	✓	IMN/Plates	93%	2 infections	Favorable outcomes
**Abbreviations:** IF = Internal Fixation; IMN = Intramedullary Nail; LCP = Locking Compression Plate; EF = External Fixator; ASAMI = Association for the Study and Application of the Method of Ilizarov; NS diff = No significant difference; NR = Not reported; LEFS = Lower Extremity Functional Scale.										

(B)										
	First Author (Year)	Design	N	Gustilo/AO	External Fixator	Converted to IF?	IF Type	Union Rate	Infection Rate	Functional Outcome
1	Lambiris (2004)^22^	Retrospective	30	Nonunion	Ilizarov	✓	IMN	∼100%	1 infection	Good union at docking site
2	Dipak (2019)^23^	Retrospective	10	Infected femur	Ilizarov (knee)	X	–	100%	Resolved	Paley: Excellent

**Abbreviations:** IF = Internal Fixation; IMN = Intramedullary Nail; LCP = Locking Compression Plate; EF = External Fixator; ASAMI = Association for the Study and Application of the Method of Ilizarov; NS diff = No significant difference; NR = Not reported; LEFS = Lower Extremity Functional Scale.

### 3.2 Quality assessment results

Overall methodological quality was moderate. Newcastle–Ottawa Scale scores ranged from 5 to 7 (median 6), reflecting frequent limitations in cohort comparability and incomplete adjustment for confounding (e.g., Gustilo grade, contamination, and soft-tissue management). Outcome ascertainment was generally adequate, but follow-up durations were variably reported and functional outcomes were inconsistently collected. Item-level NOS judgements for each domain are presented in Supplementary Table S1. Several studies enrolled selected conversion candidates, increasing the risk of selection bias across groups.

### 3.3 Infection rates

Because formal meta-analysis was not feasible, infection outcomes are presented as a narrative evidence summary rather than as study counts. In the acute-trauma comparative cohorts, reporting of deep infectious complications was inconsistent, definitions varied substantially and adjustment for confounding was limited. Some studies described fewer superficial or pin-site complications after staged conversion, whereas deep infectious outcomes remained difficult to compare reliably across designs, definitions and injury severities.

### 3.4 Nonunion, delayed union and functional outcomes

Most studies reported high overall union rates, but these findings derive largely from small retrospective cohorts and are strongly influenced by injury pattern, soft-tissue management and selection of patients for conversion. Accordingly, we do not report pooled or unweighted mean union rates and we avoid cross-study numeric averaging where formal quantitative synthesis is not justified.

### 3.5 Timing and additional procedures

Timing of conversion was inconsistently defined and variably reported across studies. Some authors described time from injury, others from provisional fixation and others from wound stabilization or soft-tissue coverage. Because definitions, clinical readiness criteria and baseline injury severity varied substantially, timing is presented descriptively only. The available literature does not support a reproducible temporal threshold for conversion and should not be interpreted as guidance for specific cutoffs.

## 4 Discussion

This systematic review provides low-certainty observational signals that staged conversion may reduce frame-related morbidity in selected patients; however, its effects on deep infectious complications and union remain uncertain because the available evidence is sparse, retrospective, and clinically heterogeneous. However, deep infection and nonunion rates did not clearly differ between strategies, and the available evidence is limited by small sample sizes, heterogeneous fracture patterns, and variable timing of conversion. Pin site infections are a well-documented drawback of prolonged ERF use, contributing to discomfort, higher reoperation rates, and the risk of osteomyelitis if neglected.^24^ By switching to intramedullary nailing or plating once soft tissue conditions improve, surgeons can often curtail superficial infection sources, promote robust alignment, and accelerate weight-bearing exercises. Our findings are broadly consistent with trauma and limb-reconstruction literature suggesting that external fixation, particularly circular ring constructs, remains valuable in complex open injuries, but its role should be individualized rather than routine. The FIXIT clinical trial in open tibial fractures did not demonstrate a clear advantage of ring external fixation over internal fixation for limb complications or infection, highlighting the importance of patient selection and soft-tissue care. Recent work on circular constructs, including Ilizarov vs monorail comparisons,^25^ reports of the ALFA device,^26^ and hinge-based Ilizarov techniques,^27^ illustrate ongoing technical evolution, yet also underscores the need for standardized outcome reporting. In the included studies, conversion timing was inconsistently defined and rarely linked to objective criteria for soft-tissue readiness or infection clearance. Several cohorts reported fewer pin-tract complications after conversion and more events with prolonged frame retention; however, these observations are vulnerable to confounding because delayed conversion often occurred in more severe injuries requiring additional procedures. Accordingly, we present timing only as described in the original studies and avoid imposing uniform intervals that could be misread as clinical guidance. Moreover, infection definitions varied and only a minority of studies applied fracture-related infection (FRI) consensus criteria, further limiting cross-study comparability and supporting our operational definitions.

From a functional standpoint, removing the external frame can facilitate more comfortable, systematic physiotherapy, thereby supporting better joint mobility and muscle conditioning.^7^

Nonetheless, variation in fracture severity, comorbidities, and local resources suggests that conversion is not a universal panacea. Definitive external fixation remains indispensable in highly contaminated or complex injuries (Gustilo IIIB/C), infected nonunions, or when patient health status precludes additional surgery.^20^^,^^23^ Hybrid approaches integrating limited internal fixation (e.g., K-wires, partial plating) with circular or monolateral frames appear to offer an effective middle ground, particularly in circumstances where full internal fixation poses elevated risks.^28^ Interpretation of infection data across studies is hampered by inconsistent terminology and variable diagnostic thresholds. Very few series applied contemporary consensus criteria for fracture-related infection, and several older reports conflated superficial pin-site inflammation with deep infection or osteomyelitis. Future studies should adopt standardized FRI definitions to permit more meaningful comparison between external and internal fixation strategies.

The evidence base remains restricted by a preponderance of retrospective designs and heterogeneous outcome measures. While union rates in both conversion and non-conversion groups often exceed 90%, the timing of conversion, the specific type of fixation device, and the presence of comorbid conditions each influence success. Economic implications also warrant exploration, as staged conversion necessitates multiple surgical events, while definitive external fixation demands diligent frame maintenance and potential corrections over time.

### 4.1 Limitations

This review has several important limitations. First, most included studies were retrospective, single-center cohorts at moderate risk of selection and confounding bias, with very few randomized data. Second, there was substantial clinical heterogeneity in fracture location, Gustilo–Anderson grade, soft-tissue management, and timing and type of internal fixation, which limited the scope and interpretability of quantitative analyses. Third, several included series mixed open and closed fractures or reconstructive indications with acute trauma, and reporting of comorbidities, smoking status, and microbiological data was inconsistent. Finally, the small number of studies per outcome precluded robust exploration of publication bias and prevented formal grading of the certainty of evidence. Therefore, the present review should be interpreted as a descriptive synthesis of heterogeneous observational evidence and should not be used to infer causal effects or a universal “safe” timing threshold for conversion after ring fixation.

### 4.2 Future directions

Moving forward, standardized protocols that clearly define objective criteria for soft-tissue recovery, infection clearance and patient readiness would be valuable. Large-scale, multicenter prospective studies comparing definitive ring fixation versus staged conversion using clearly reported clinical readiness criteria would help clarify the effect of conversion on infection and functional outcomes.

## 5 Conclusions

In adults with acute traumatic open long-bone fractures managed with circular ring external fixation, the current literature provides low-certainty observational signals rather than robust comparative evidence regarding staged conversion to internal fixation. Staged conversion may reduce frame-related morbidity and facilitate rehabilitation in selected cases after adequate soft-tissue recovery but its effects on deep infectious complications and union cannot be defined reliably from the available retrospective and highly heterogeneous literature. Accordingly, decisions regarding conversion should remain individualized. Future prospective studies should use consistent infection and healing definitions, objective readiness criteria, and clearly prespecified descriptive reporting of conversion timing.

## Informed consent

Not applicable.

## Declaration of ethical approval for study

Does not required.

## Declaration of informed consent

There is no information (names, initials, hospital identification numbers, or photographs) in the submitted manuscript that can be used to identify patients.

## Authors’ contributions

✓ Authors who conceived and designed the analysis: Gabriele Giuca, Domenico Fenga.
✓ Authors who collected the data: Gabriele Giuca, Domenico Fenga, Paolo Rizzo.
✓ Authors who contributed data or analysis tools: Daniel Pérez-Prieto, Paolo Rizzo.
✓ Authors who performed the analysis: Domenico Fenga, Biagio Zampogna
✓ Authors who wrote the paper: Domenico Fenga, Gabriele Giuca.
✓ Other contribution: Danilo Leonetti and Daniel Pérez-Prieto reviewed the paper

## Ethical approval

Not required as this study is a systematic review of published data.

## Declaration of generative AI and AI-assisted technologies in the writing process

The authors declare that no generative AI or AI-assisted technologies were used in the writing process of this manuscript.

## Funding

No specific funding was received for this work.

## Declaration of competing interest

The authors declare that they have no known competing financial interests or personal relationships that could have appeared to influence the work reported in this paper.

## References

[1] Gustilo R.B., Anderson J.T. (1976). Prevention of infection in the treatment of one thousand and twenty-five open fractures of long bones: retrospective and prospective analyses. J Bone Joint Surg Am.

[2] Foote C.J., Tornetta P., Reito A. (2021). A reevaluation of the risk of infection based on time to debridement in open fractures. J Bone Joint Surg.

[3] Metsemakers W.J., Morgenstern M., McNally M.A. (2018). Fracture-related infection: a consensus on definition from an international expert group. Injury.

[4] Whiting P.S., Obremskey W., Johal H., Shearer D., Volgas D., Balogh Z.J. (2024). Open fractures: evidence-based best practices. OTA Int.

[5] Papakostidis C., Kanakaris N.K., Pretel J., Faour O., Morell D.J., Giannoudis P.V. (2011). Prevalence of complications of open tibial shaft fractures stratified as per the Gustilo-Anderson classification. Injury.

[6] Giannoudis P.V., Papakostidis C., Roberts C. (2006). A review of the management of open fractures of the tibia and femur. J Bone Joint Surg Br.

[7] Paley D. (1990). Problems, obstacles, and complications of limb lengthening by the Ilizarov technique. Clin Orthop Relat Res.

[8] Monni T., Birkholtz F.F., de Lange P., Snyckers C.H. (2013). Conversion of external fixation to internal fixation in a non-acute, reconstructive setting: a case series. Strategies Trauma Limb Reconstr.

[9] Pape H.C., Tornetta P., Tarkin I., Tzioupis C., Sabeson V., Olson S.A. (2009). Timing of fracture fixation in multitrauma patients: the role of early total care and damage control surgery. J Am Acad Orthop Surg.

[10] Dhar S.A., Butt M.F., Hussain A., Mir M.R., Halwai M.A., Kawoosa A.A. (2008). Management of lower limb fractures in polytrauma patients with delayed referral in a mass disaster. The role of the Ilizarov method in conversion osteosynthesis. Injury.

[11] Wynn M.S., Jang Y., Ochenjele G., Natoli R.M. (2024). External fixation before planned conversion to internal fixation in orthopaedic trauma: controversies and current trends. J Am Acad Orthop Surg.

[12] Ye Z., Zhao S., Zeng C., Luo Z., Yuan S., Li R. (2021). Study on the relationship between the timing of conversion from external fixation to internal fixation and infection in the treatment of open fractures of extremities. J Orthop Surg Res.

[13] Megas P., Saridis A., Kouzelis A., Kallivokas A., Mylonas S., Tyllianakis M. (2010). The treatment of infected nonunion of the tibia following intramedullary nailing by the Ilizarov method. Injury.

[14] Burg A., Berenstein M., Engel J. (2011). Fractures of the distal humerus in elderly patients treated with a ring fixator. Int Orthop.

[15] Santolini E., Giordano V., Giannoudis P.V. (2024). Effect of mechanical stability of osteosynthesis on infection rates: timing of temporary and definitive fixation. Injury.

[16] Aljuhani W.S., Alshabi Y.A., Alanazi A.M. (2024). Risk of infection and conversion time from external to definitive fixation in open tibial fracture. J Orthop Surg Res.

[17] Zhang D.H., Lei G., Yi L., Wang Y., Li Y.S., Wen H.J. (2020). Free flap transfer combined with the Ilizarov technique for type IIIB and IIIC open fracture of extremities: experience with 8 cases. J Orthop Transl.

[18] Lambiris E., Papadopoulos A.X., Karabasi A., Karageorgos A. (2004). Secondary intramedullary nailing after distraction osteogenesis: 30 patients followed for 2-12 years. Acta Orthop Scand.

[19] Sala F., Elbatrawy Y., Thabet A.M., Zayed M., Capitani D. (2012). Taylor spatial frame fixation in patients with multiple traumatic injuries: study of 57 long-bone fractures. http://links.lww.com/BOT/A90.

[20] Bacon S., Smith W.R., Morgan S.J. (2008). A retrospective analysis of comminuted intra-articular fractures of the tibial plafond: open reduction and internal fixation versus external Ilizarov fixation. Injury.

[21] Kumar Jha Dipak, Sunny Kumar Mallick (2019). The use of the knee spanning Ilizarov method as a treatment procedure in infected nonunion of the distal femur with bone loss. Genij Ortopedii.

[22] Jin L., Zhang S., Zhang Y., Lin X., Feng D., Hu K. (2022). Management algorithm of external fixation in lower leg arterial injury for limb salvages. BMC Surg.

[23] Lim J.A., Thahir A., Zhou A.K., Girish M., Krkovic M. (2020). Definitive management of open pilon fractures with fine wire fixation. Injury.

[24] O'Toole R.V., Gary J.L., Reider L. (2017). A prospective randomized trial to assess fixation strategies for severe open Tibia fractures: modern ring external fixators versus internal fixation (FIXIT study). J Orthop Trauma.

[25] Maheshwari V., Raja B.S., Bahadur B., Regmi A., Dhingra M., Gowda A.K.S. (2023). Outcome analysis of ilizarov and monorail fixators in the treatment of nonunion of long bones: a systematic review and proportion meta-analysis. J Clin Orthop Trauma.

[26] Vardhan S., Regmi A., Niraula B.B., Kunwar B.B., Olkha V., Dhingra M. (2024). Articulated lengthening fixation apparatus (ALFA) for the management of gap non-union of distal femur: initial experience on a new technique. J Orthop.

[27] Regmi A., Niraula B.B., Bansal S., Gowda R., Barman S., Dhingra M. (2023). Gradual distraction on hinged-based Ilizarov ring fixator to correct fixed flexion deformity of knee: description of the surgical technique. Int J Res Orthop.

[28] Liang H., Li L., Yang J., Du Y., Peng W. (2021). Treatment of open and comminuted mid-distal tibial fractures by bilateral external fixation combined with limited-internal fixation. Acta Orthop Belg.

