# Supplementary material for: Staged conversion after circular ring external fixation in adult open long-bone fractures: A systematic review
Source: J Clin Orthop Trauma. 2026 Mar 6;75:103398. doi: 10.1016/j.jcot.2026.103398 (PMC12993332; doi:10.1016/j.jcot.2026.103398)
Supplement: Supplementary file 1 [file mmc1.docx]

**Supplementary Table S1.** Risk of bias assessment of included studies using the Newcastle–Ottawa Scale (NOS). The NOS evaluates cohort studies across three domains: **Selection** (representativeness of the exposed cohort; selection of the non-exposed cohort; ascertainment of exposure; demonstration that the outcome of interest was not present at the start of the study), **Comparability** (control for the most important confounder and for an additional confounder), and **Outcome** (assessment of outcome; adequacy of follow-up duration; adequacy of follow-up completeness). A star (★) indicates that the study met the criterion; a dash (–) indicates that the criterion was not met. **NA** denotes items not applicable to **single-arm cohorts** (i.e., absence of a non-exposed comparator cohort and inability to assess comparability). For these studies, non-applicable items were excluded from the denominator when reporting total NOS scores (**maximum 6 stars** for single-arm studies; **maximum 9 stars** for comparative cohorts). NOS, Newcastle–Ottawa Scale.

| **Study** | **Design** | **Population** | **Fixator** | **NOS Rep** | **NOS Non-Exp** | **NOS Asc** | **NOS Outcome absent** | **NOS Comp (1)** | **NOS Comp (2)** | **NOS Outcome assess** | **NOS Follow-up** | **NOS Adequacy** | **Total (achieved/applicable)** | **Notes** |
| --- | --- | --- | --- | --- | --- | --- | --- | --- | --- | --- | --- | --- | --- | --- |
| Zhang (2020)^17^ | Retrospective | Open fractures IIIB–IIIC | Ilizarov | ★ | NA | ★ | ★ | NA | NA | ★ | ★ | — | 5/6 | Single-arm cohort/case series |
| Lambiris (2004)^18^ | Retrospective | Open tibial fractures | Ilizarov | ★ | NA | ★ | ★ | NA | NA | ★ | ★ | — | 5/6 | Single-arm cohort/case series |
| Sala (2013)^19^ | Retrospective | Open long bone fractures | Taylor Spatial Frame | ★ | NA | ★ | ★ | NA | NA | ★ | ★ | — | 5/6 | Single-arm cohort/case series |
| Dhar (2008)^10^ | Descriptive | Open long bone fractures | Ilizarov | ★ | NA | ★ | ★ | NA | NA | ★ | ★ | — | 5/6 | Single-arm cohort/case series |
| Bacon (2008)^20^ | Retrospective | Type C pilon fractures | Ilizarov vs ORIF | ★ | ★ | ★ | ★ | ★ | — | ★ | ★ | — | 7/9 | Comparative cohort. |
| Dipak (2019)^21^ | Retrospective | Infected nonunion distal femur (post-trauma) | Ilizarov (knee-spanning) | ★ | NA | ★ | — | NA | NA | ★ | ★ | — | 4/6 | Infected nonunion at baseline. Single-arm cohort/case series. |
| Jin (2022)^22^ | Retrospective | Open long bone fractures w/ arterial injury | Mixed EF | ★ | NA | ★ | ★ | NA | NA | ★ | ★ | — | 5/6 | Single-arm cohort/case series |
| Lim (2020)^23^ | Retrospective | Open long bone fractures | Monolateral EF | ★ | NA | ★ | ★ | NA | NA | ★ | ★ | — | 5/6 | Single-arm cohort/case series |
